# 

**Published:** 1882-08

**Authors:** 


P. W. VAN PEYMA, M. D.,

INDEX MEDICUS.

### CONTENTS:

| | PAGE. |
|---|---|
| ORIGINAL COMMUNICATIONS: | |
| Spinal Extension by Suspension, By Bernard Bartow, M. D. | 1 |
| The Present Status of Medicine and Its Relation to the Commonwealth in the Future. By F. J. Baker, M. D, | 7 |
| A Case Illustrating the Importance of Diagnosis. By H. R. Hopkins. M. D., | 14 |
| CLINICAL REPORTS: | |
| Complete Placenta Prævia. By P. W. Van Peyma, M. D., | 16 |
| Delivery at Six Months with Membranes Unruptured. By P. W Van Peyma, M. D., | 17 |
| SELECTIONS: | |
| The Bromides in Inebriety, | 18 |
| Malaria and Diabetes, | 19 |
| Dialyzed Iron in the Treatment of Arsenical Poisoning, | 21 |
| Treatment of Acne, | 23 |
| Practical Notes on Neuralgia and its Treatment, | 29 |
| Coated Pills, | 31 |
| Sulphide of Calcium as an Antisuppurative, | 32 |
| Billroth on Removal of Cancer, | 33 |
| CORRESPONDENCE: | |
| Letter from Strassburg. By Frederick Peterson, | 34 |
| "Sewer Gas and its Dangers," Letter from Prof. F. H. Hamilton, | 37 |
| SOCIETY REPORTS: | |
| Buffalo Medical and Surgical Association, | 39 |
| EDITORIAL: | |
| Volume XXII, | 42 |
| The Buffalo Medical College, | 43 |
| Editorial Changes, | 45 |
| Editorial Notes, | 45 |
| REVIEWS: | 47 |

